# Alterations in structural integrity of superior longitudinal fasciculus III associated with cognitive performance in cerebral small vessel disease

**DOI:** 10.1186/s12880-024-01324-2

**Published:** 2024-06-10

**Authors:** Yifan Wang, Tianyao Wang, Zekuan Yu, Junjie Wang, Fang Liu, Mengwen Ye, Xianjin Fang, Yinhong Liu, Jun Liu

**Affiliations:** 1 Department of Radiology, Eye& ENT Hospital of Shanghai Medical School, Fudan University, Shanghai, China; 2 Department of Radiology, Renji Hospital, Shanghai Jiao Tong University School of Medicine, Shanghai, China; 3 Academy for Engineering and Technology, Fudan University, Shanghai, 200433 China; 4 Department of Neurosurgery, Beijing Hospital, National Center of Gerontology, Beijing, China; 5 Institute of Geriatric Medicine, Chinese Academy of Medical Sciences, Beijing, China; 6 Department of Neurology, Beijing Hospital, National Center of Gerontology, Beijing, China; 7 Anhui University of Science and Technology, Anhui, China; 8 Department of Radiology, Tongren Hospital, Shanghai Jiao Tong University School of Medicine, 1111 XianXia Road, Shanghai, 200050 China

**Keywords:** Cerebral small vessel disease, White matter hyperintensities, Diffusion tensor imaging, Superior longitudinal fasciculus, Cognition

## Abstract

**Background:**

This study aimed to investigate the alterations in structural integrity of superior longitudinal fasciculus subcomponents with increasing white matter hyperintensity severity as well as the relationship to cognitive performance in cerebral small vessel disease.

**Methods:**

110 cerebral small vessel disease study participants with white matter hyperintensities were recruited. According to Fazekas grade scale, white matter hyperintensities of each subject were graded. All subjects were divided into two groups. The probabilistic fiber tracking method was used for analyzing microstructure characteristics of superior longitudinal fasciculus subcomponents.

**Results:**

Probabilistic fiber tracking results showed that mean diffusion, radial diffusion, and axial diffusion values of the left arcuate fasciculus as well as the mean diffusion value of the right arcuate fasciculus and left superior longitudinal fasciculus III in high white matter hyperintensities rating group were significantly higher than those in low white matter hyperintensities rating group (*p* < 0.05). The mean diffusion value of the left superior longitudinal fasciculus III was negatively related to the Montreal Cognitive Assessment score of study participants (*p* < 0.05).

**Conclusions:**

The structural integrity injury of bilateral arcuate fasciculus and left superior longitudinal fasciculus III is more severe with the aggravation of white matter hyperintensities. The structural integrity injury of the left superior longitudinal fasciculus III correlates to cognitive impairment in cerebral small vessel disease.

**Supplementary Information:**

The online version contains supplementary material available at 10.1186/s12880-024-01324-2.

## Introduction

Cerebral small vessel disease (CSVD) refers to a group of clinical syndromes resulting from pathologies in cerebral arterioles, microarteries, capillaries, and venules, which is responsible for approximately 20% of ischemic stroke worldwide [1]. It is considered to be the leading cause of vascular cognitive impairment (VCI) [2]. White matter hyperintensity (WMH) is one of the typical radiological features of CSVD, which has been reported to be related to the occurrence and progression of cognitive decline [3–5]. Nevertheless, the pathogenesis of WMH-related cognitive dysfunction in CSVD remains to be further explored. In effect, a routine sequence of magnetic resonance imaging (MRI) can only roughly distinguish normal tissue from abnormal tissue, which fails to reflect the range along with the severity of white matter structural integrity injury accurately [6]. Diffusion tensor imaging (DTI) is an advanced technique that plays a vital role in detecting microstructural changes in white matter [7–9]. Utilizing DTI technology, several researchers have found that changes in white matter structural integrity in CSVD study participants are related to cognitive function [10–15]. Hu et al. pointed out that CSVD study participants own decreased fractional anisotropy (FA) value along with increased mean diffusion (MD), radial diffusion (RD), and axial diffusion (AD) values of some white matter fiber tracts compared to normal subjects, which is associated with cognitive impairment [16]. Liu et al. demonstrated that the integrity of extensive white matter (WM), such as the corpus callosum, superior longitudinal fasciculus (SLF), and inferior longitudinal fasciculus, has already been damaged at the pre-CSVD stage, which would be associated with future cognitive dysfunction [17]. Du et al. indicated that structural integrity changes of some specific longitudinal tracts such as SLF, superior fronto-occipital fasciculus, and uncinate fasciculus involving the frontal-parietal–subcortical network have been captured in the preclinical stage of VCI, which might add more knowledge to the underlying pattern of white matter disruption in CSVD [18].

Among all white matter fibers, the SLF connects the frontal, occipital, parietal together with temporal lobes, which is known as a complex association fiber tract [19]. The SLF contributes to forming a bidirectional neural network, which plays an important role in terms of attention, memory as well as language [20, 21]. There are four subcomponents of SLF, including SLF I, SLFII, SLFIII, and arcuate fasciculus (AF) [22]. Each SLF subcomponent owns unique function [23]. SLF I is mainly involved in attentional functions such as top-down attention, voluntary orientation of attention, and working memory. SLF II involves motor control and executive function, including working memory. The most important function of SLF III is related to a language network, involving articulatory processing and auditory language comprehension. The function of AF is related to the visuospatial attentional network. It is involved in social cognition, language, and phonology. Previous studies have suggested that impairment of the structural integrity of SLF is related to cognitive impairment in CSVD study participants [24, 25]. Nevertheless, little is known about alterations in the structural integrity of four subcomponents of SLF with increasing WMH severity and their relationship with cognitive decline in CSVD study participants.

In this article, we recruited a group of CSVD study participants with WMH findings on MRI. Probabilistic fiber tracking analysis was used to extract the diffusion measurements of each SLF subcomponent to capture the microstructure characteristics of each subcomponent. We hypothesized that as the severity of WMH increased, the diffusion measurements in some subcomponents of the SLF are different, and these different diffusion measurements are related to different cognitive performance in CSVD study participants.

## Materials and methods

### Participants

**Fig. 1:**
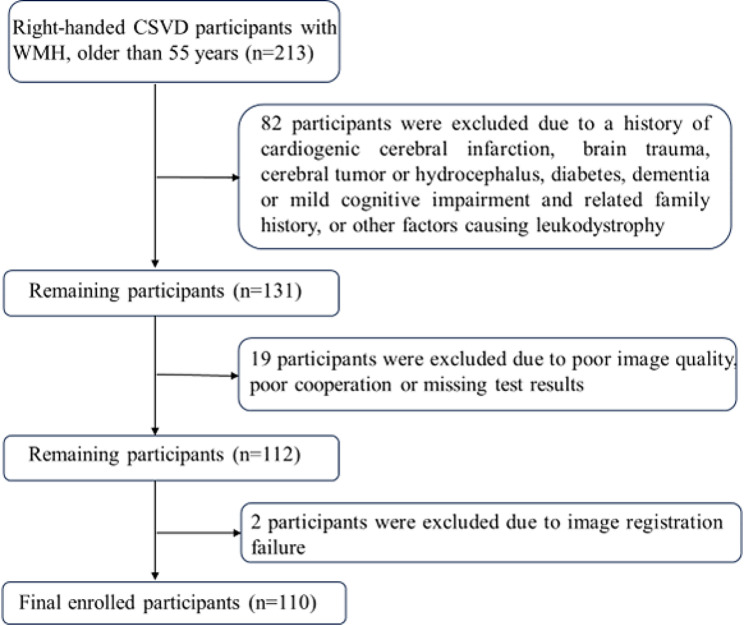
The flowchart of study 110 participants. CSVD, cerebral small vessel disease; WMH, white matter hyperintensity;

The clinical manifestations of CSVD lack specificity, and the diagnosis mainly depends on imaging examination. A convenient method for clinical application is MRI total score of CSVD. One point was awarded for each of the following items: moderate to extensive perivascular spaces in the basal ganglia (1 point if present); ≥1 asymptomatic lacune (1 point if present); periventricular WMH Fazekas score 3 or if deep WMH Fazekas score 2 or 3 (1 point if present); ≥1 deep cerebral microbleed [3]. All imaging characterizations follow the definition of Standards for Reporting Vascular Changes on Neuroimaging 2 (STRIVE-2) [26]. When the total score is greater than or equal to 2 points, the diagnosis of CSVD is considered. In this study, only WMH of CSVD was investigated. The inclusion criteria include the following points: (1) Right-handed participants aged at least 55 years old, (2) meet at least two of the following conditions: (1) moderate to extensive perivascular spaces in basal ganglia, (2) at least one asymptomatic lacune, (3) deep WMH Fazekas score of 2 or 3 points or periventricular WMH Fazekas score of 3 points, (4) at least one deep cerebral microbleed, (3) bilateral hyperintensities in periventricular along with deep white matter regions on T2-weighted as well as fluid attenuation inversion recovery (FLAIR) sequences. The exclusion criteria include the following points: (1) a history of ischemic stroke with the ischemic lesion diameter of over 15 mm, (2) a history of cardiogenic cerebral infarction, (3) a history of brain trauma, (4) a history of cerebral tumor or hydrocephalus, (5) other factors causing leukodystrophy (such as multiple sclerosis, a history of brain exposure and so on), (6) a history of diabetes, (7) a history of dementia or mild cognitive impairment and related family history. Study participants with poor image quality, poor cooperation, missing test results, and image post-processing failure were excluded. In the end, a total of 110 CSVD participants with WMH were included in the current study. See Fig. 1 for details. The mean age of these participants was 67.3 ± 6.55 years, including 33 males and 77 females. None of the participants had a clear clinical diagnosis of amyloid-associated, hereditary, inflammation or immune-mediated, post-radiation CVSD or venous collagenosis [27]. 

### Data collection as well as cognitive assessment

In this study, the demographic data of each study participant were recorded in detail, including gender, age, education level as well as clinical history. The blood biochemical test results of each patient were tracked. In addition, all study participants underwent structural and DTI sequences MR scans. Later, two radiologists checked the image quality of the MR scan strictly [27]. 

All study participants underwent cognitive assessments within a week of the MR examination. To test the global cognitive function of study participants, we adopted the Mini-mental State Examination (MMSE) as well as the Montreal Cognitive Assessment (MoCA) scales. The total test scores for each study participant were recorded [27]. 

The current study was conducted in line with The Code of Ethics of the World Medical Association (Declaration of Helsinki), and it was approved by the research ethics committee of Tongren Hospital, Shanghai Jiao Tong University School of Medicine. The informed consent form had been signed by each subject.

### MRI acquisition

MRI scanning was performed on Siemens 3 Tesla Skyra scanner (Siemens, Germany) with 20 channel head-neck coil at Shanghai Fifth People’s hospital. The 3D T1-weighted image were used MPRAGE sequence as follow parameters: TR = 2400ms, TE = 2.13ms, TI = 1100ms, FOV = 256 × 256mm^2^, thickness = 1 mm, voxel size = 1 × 1 × 1mm^3^, and number of slices = 192. The scan parameters of 3D T2W-FLAIR were as follows: TR = 5000ms, TE = 395ms, TI = 1800ms, FOV = 256 × 256mm^2^, thickness = 1 mm, voxel size = 1 × 1 × 1mm^3^, and number of slices = 192. The DTI were used SE-EPI sequence as follows: TR = 8300ms, TE = 74ms, FOV = 256 × 256mm^2^, thickness = 2 mm, voxel size = 2 × 2 × 2mm^3^, number of slices = 72, 30 diffusion weighted images of b = 1000s/mm^2^ and another five b0 images were acquired.

### Image processing

Image processing mainly includes the following steps [28, 29]: (1) Visually check all DTI data in mosiac format via PACS and delete the data affected with inter slice brightness abnormal even slice miss inter volume, then convert dicom to NIFTI via dcm2niix and remove all images affected by missing coverage whole brain and obvious motion artifacts. (2) Extract the first B0 images and skull stripped using the BET2 tool of FSL for data quality control (QC). (3) Perform automatic QC protocol with DTIprep (v1.2.11), and delete the no passed data. (4) Complete head movement and eddy current correction with eddy correct of FSL. (5) Extract all B0 images and get the mean B0 image, skull stripped using the BET2 tool. Check brain BET quality for each data and correct the error mask manually with ITKSNAP. (6) Fit diffusion tensor results such as fractional anisotropy (FA), mean diffusivity (MD), radial diffusivity (RD), and axial diffusivity (AD) on eddy-corrected data with DTIFIT. Check all results manually for FA and V1 images and delete the data with obvious errors on the V1 RGB image. (7) Skull stripped using BET2 tool for 3D-T1WI images. Check brain BET quality for each data and correct the error mask manually with ITKSNAP. (8) Registration diffusion image to MNI152 standard space with 3D-T1WI structural image via FLIRT and FNIRT. Check all warped images and delete error data. (9) Run GPU version bedpostx and XTRACT for probabilistic fiber tractography. Visually check all fiber results in standard space and remove error data. Then export all results to csv file for post-statistical analysis. Finally, the volume, length, FA, MD, AD, and RD values of the AF, SLFI, SLFII, and SLFIII were obtained. See the analysis code excerpts and some MRI processing figures in Supplementary Material 1 and 2. In this study, a total of 2 subjects with diffusion image registration failure were excluded.

### WMH grading evaluation

One junior radiologist with three years of experience and one senior radiologist with twelve years of experience evaluated the WMH severity independently by observing the MR FLAIR sequence image without knowing the clinical data. WMH located in periventricular along with deep white matter were graded separately according to the Fazekas grade scale (Table 1) [30]. And then, radiologists added the two grades together to get the total score. Zeng demonstrated that study participants began to show significant white matter microstructure injury when Fazekas score of WMH reached 3 points [31]. While no significant difference was found in terms of microstructure integrity between the mild WMH group and the non-WMH group [31]. Hence, we divided all CSVD study participants with WMH into two groups. In the WMH low rating group (Group A), study participants own 1–2 points of the WMH score. In the WMH high rating group (Group B), study participants own 3–6 points of the WMH score. Intra reliability for the junior radiologist with three years of experience was assessed on 30 randomly selected WMH images scored twice and turned out to be good with a Kappa coefficient of 0.927. Inter reliability for the junior radiologist (three years of experience) and senior radiologist (twelve years of experience) turned out to be good with Cronbach’s α value of 0.892. When two radiologists met disagreement, they discussed and reevaluated it, and finally, the evaluation result of the senior radiologist (twelve years of experience) was accepted.

In addition, we also used the age-related white matter changes (ARWMC) scale [32] to assess the severity of WMH, as detailed in Supplementary Material 3. The Wisconsin White Matter Hyperintensities Segmentation Toolbox (W2MHS) (http://www.nitrc.org/projects/w2mhs) was used to automatically mark and extract the volume of the WMH. See Supplementary Material 4 for details.

**Table 1:** Fazekas grade scale

Grade	Periventricular hyperintensity (PVH)	Deep white matter hyperintense signals (DWMH)
0	absence	absence
1	caps or pencil-thin lining	punctate foci
2	Smooth halo	beginning confluence of foci
3	irregular PVH extending into the deep white matter	large confluent areas

Wang Y, Liu X, Hu Y, Yu Z, Wu T, Wang J, Liu J, Liu J. Impaired functional network properties contribute to white matter hyperintensity related cognitive decline in patients with cerebral small vessel disease. BMC MED IMAGING 2022;22:40. The current article included the same subjects and grouping criteria as the previously published article. According to the regulations of BMC Medical Imaging, as an author of an article published in BMC Medical Imaging, we retain the copyright of our article and we are free to reproduce and disseminate our work.

### Statistical analysis

SPSS26.0 statistical software was applied for statistical analysis. T-test, chi-square test, and nonparametric test were used to analyze demographic, clinical characteristics along with cognitive assessment data. T-test was used to analyze the difference in the volume, length, FA, MD, AD, and RD values of the four subcomponents of SLF between the two groups. False discovery rate (FDR) correction is adopted to control class I errors. Age, gender, and education level corrections were achieved by multiple linear regression analysis. *P* < 0.05 was considered statistically significant. To evaluate the relationship between different DTI-derived indexes and global cognitive function, partial correlation analysis was applied. In partial correlation analysis, we considered age, gender along with education level as covariant. We also used partial correlation analysis to explore the relationship between WMH volume and DTI-derived indexes, with age, sex, and education as covariates.

## Results

### Demographic, clinical features, and neuroimaging information

Other than age, there was no statistical difference between groups on the aspect of demographic data. The mean age of study participants in the WMH high rating group was older than that of study participants in the WMH low rating group. See Table 2 for more details. In this study, 110 patients had a median ARWMC score of six, with an interquartile range of three to twelve. See Table S2 for the distribution of WMH in study participants.

**Table 2:** Demographic, clinical characteristics, and cognitive assessment data

Items	Group A(*n* = 64)	Group B(*n* = 46)	t-value	*p*-value
Age	65(7)	69(13)	3.769	0.001**
Female, n(%)	44(68.7)	33(71.1)	1.264	0.736
Hypertension, n (%)	24(37.5)	13(31.7)	1.005	0.264
Hyperlipemia, n(%)	31(56.4)	20(57.1)	1.234	0.752
TC	4.52(1.08)	4.38 ± 0.16	0.883	0.586
TG	1.21(0.64)	1.50(0.98)	1.075	0.199
HDL	1.37 ± 0.56	1.27 ± 0.69	1.093	0.140
LDL	2.85 ± 0.10	2.61(1.42)	1.064	0.276
MMSE	29.00(1.00)	29.00(2.00)	1.089	0.091
MOCA	24.00(5.00)	24.00(5.00)	1.080	0.095

Values with normal distribution are presented as the mean ± stand deviation (SD); Values with non-normal distribution are presented as median (interquartile range)

TC: total cholesterol; TG: triglyceride; HDL: high density lipoprotein; LDL: low density lipoprotein; MMSE: mini-mental state examination; MoCA: montreal cognitive assessment; The Group A = WMH low rating group. The Group B = WMH high rating group

*: The difference between groups was statistically significant(*p* < 0.05)

Wang Y, Liu X, Hu Y, Yu Z, Wu T, Wang J, Liu J, Liu J. Impaired functional network properties contribute to white matter hyperintensity related cognitive decline in patients with cerebral small vessel disease. BMC MED IMAGING 2022;22:40. The current article included the same subjects and grouping criteria as previously published article. According to the regulations of BMC Medical Imaging, as an author of an article published in BMC Medical Imaging, we retain the copyright of our article and we are free to reproduce and disseminate our work

### Group differences in DTI-derived indexes of SLF subcomponents

**Fig. 2:**
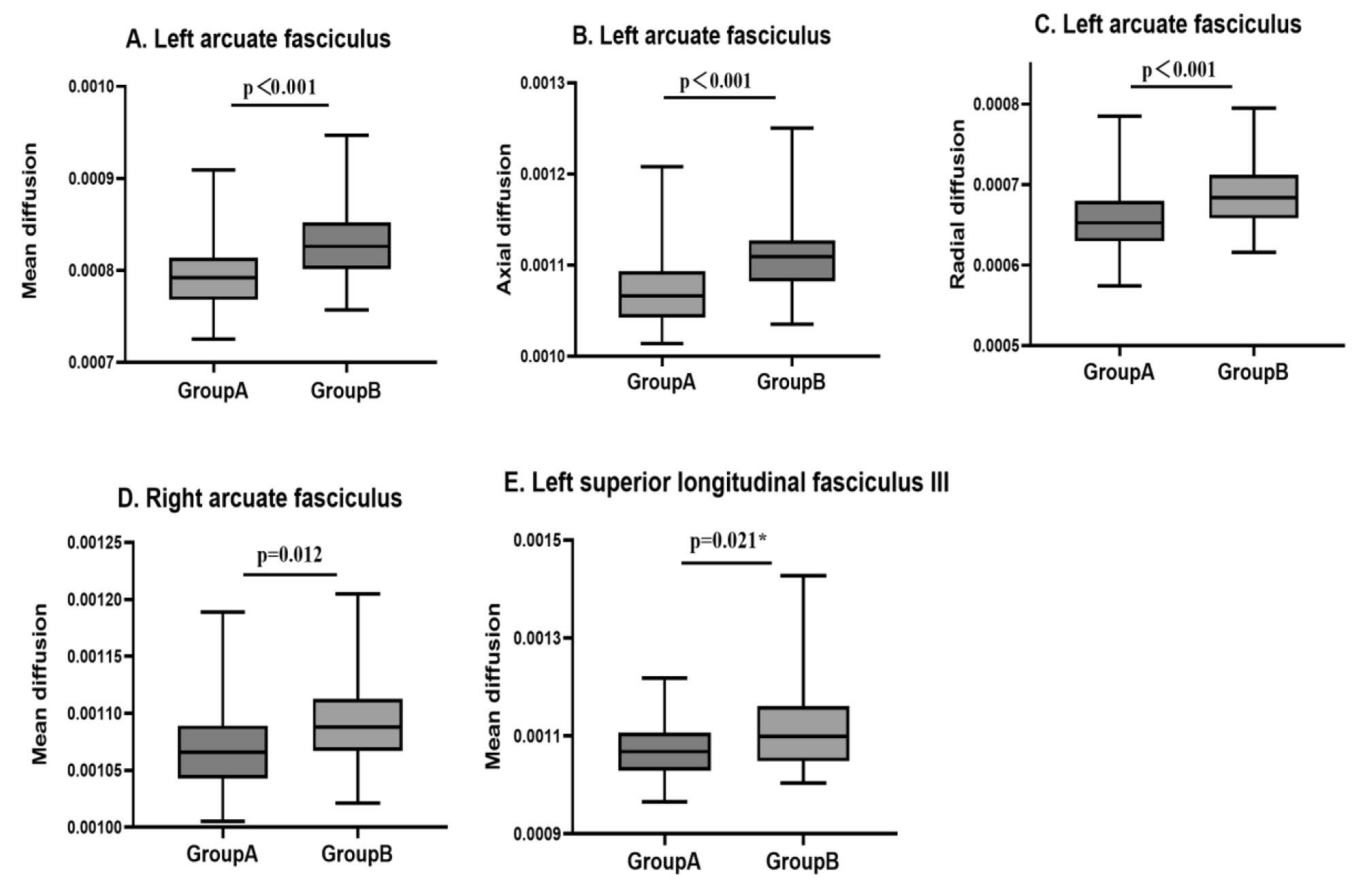
Different DTI-derived indexes of SLF subcomponents between groups

The results of probabilistic fiber tracking showed that MD, AD, and RD values of left AF as well as MD value of right AF and left SLF-III in the WMH high rating group were significantly higher than those in the WMH low rating group (*p* < 0.05, FDR corrected). No significant differences exist in fiber length and volume of the four subcomponents of SLF between groups (*p*>0.05, FDR corrected). All of these results are shown in Table 3; Fig. 2.

**Table 3:** Different DTI-derived indexes of SLF subcomponents between groups

Tract	*p*-value (volume)	*p*-value (Mean length)	*p*-value (Mean FA)	*p*-value (Mean MD)	*p*-value (Mean AD)	*p*-value (Mean RD)
SLFI-L	*p* = 0.866	*p* = 0.793	*p* = 0.703	*p* = 0.249	*p* = 0.240	*p* = 0.297
SLFI-R	*p* = 0.871	*p* = 0.902	*p* = 0.623	*p* = 0.801	*p* = 0.618	*p* = 0.822
SLFII-L	*p* = 0.463	*p* = 0.553	*p* = 0.343	*p* = 0.060	*p* = 0.054	*p* = 0.182
SLFII-R	*p* = 0.833	*p* = 0.692	*p* = 0.330	*p* = 0.148	*p* = 0.181	*p* = 0.269
SLFIII-L	*p* = 0.154	*p* = 0.213	*p* = 0.161	*p* = 0.021^*^	*p* = 0.069	*p* = 0.156
SLFIII-R	*p* = 0.358	*p* = 0.268	*p* = 0.891	*p* = 0.069	*p* = 0.120	*p* = 0.128
AF-L	*p* = 0.128	*p* = 0.109	*p* = 0.160	*p*<0.001^*^	*p*<0.001^*^	*p*<0.001^*^
AF-R	*p* = 0.517	*p* = 0.678	*p* = 0.357	*p* = 0.012^*^	*p* = 0.052	*p* = 0.156

SLF: superior longitudinal fasciculus; AF: arcuate fasciculus;

*: The difference between groups was statistically significant(*p* < 0.05, FDR corrected)

The Group A = WMH low rating group. The Group B = WMH high rating group.

### Relationship between DTI-derived indexes and cognitive performance

**Fig. 3:**
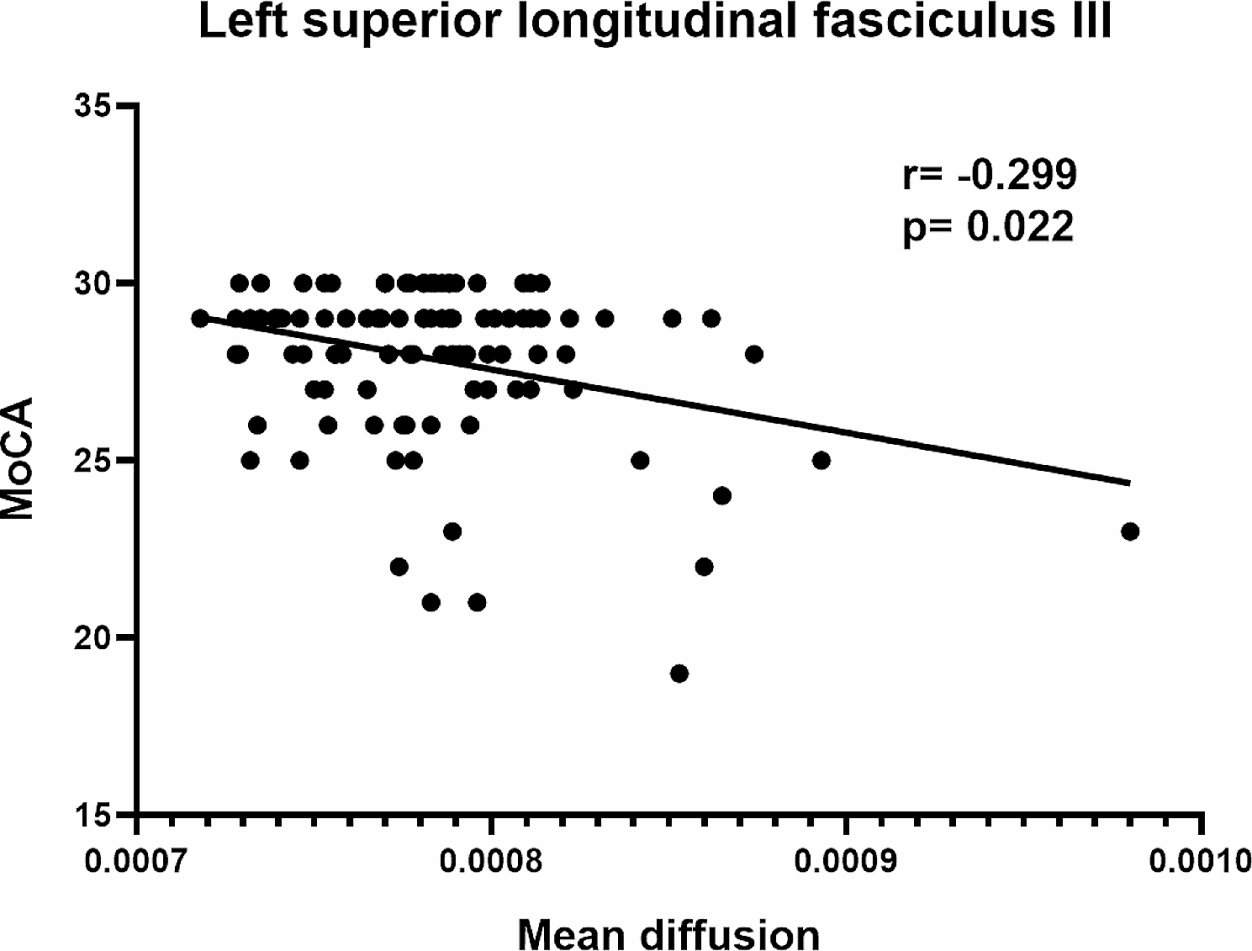
The correlation between the MD value of left SLFIII and MoCA score

We found the MD value of left SLFIII was negatively correlated with study participants’ MoCA score after controlling for age, sex, and education (*r* = 0.299, *p* = 0.022). See Fig. 3 for details.

### Relationship between WMH volume and DTI-derived indexes

The results showed that MD, AD, and RD values of bilateral AF and left SLF-III were positively correlated with the WMH volume of patients (*p* < 0.05, FDR corrected). See Table S3 in Supplementary Material 4 for details.

## Discussion

Pathological examination of WMH in CSVD showed varying degrees of axon loss, myelin thinning, ependymal disruption, and fluid accumulation, with more extensive abnormalities in more severe lesions [33]. ARWMC score results in our study showed that WMH in CSVD patients tended to occur in the frontal and parietal occipital regions, which was consistent with previous studies [34]. Several researchers have noticed that structural integrity injury of WM fiber tracts in CSVD were widely and symmetrically distributed in bilateral cerebral hemispheres [16, 17, 35]. Among these injured white matter regions, SLF is one of the vulnerable long association fibers [18]. The reason is that long association fibers mostly distribute in the periventricular region, and the blood supply of the periventricular region comes from the terminal branches of subependymal arteries which lack collateral circulation [36]. In addition, long association fiber is characterized by high energy consumption. All of these make them particularly sensitive to microvascular pathological changes [37]. Changes such as axonal injury and demyelination continue along the long association fibers, which contribute to the progression of WMH [37].

In the current study, MD, AD, and RD values of left AF as well as MD value of right AF and left SLF-III in the WMH high rating group were significantly higher than those in the WMH low rating group. According to previous research, increased MD, AD, and RD values suggest injury associated with axonal injury and demyelination, which serve as early markers of demyelination in WM regions [38–40]. This result indicates that the structural integrity injury of bilateral AF and left SLFIII was significantly more severe with the aggravation of WMH. The AF is classically known as the longest subcomponent of the SLF, which connects the temporal, parietal, and frontal language regions [41]. The SLF III is a ventral segment of SLF, which starts from the supramarginal gyrus (SMG) and terminates primarily in prefrontal and ventral premotor areas [42]. It thus appears that the longest subcomponents and the ventral segment of the SLF are more susceptible to ischemia and show more pronounced microstructure injury than other subcomponents of SLF, thus contributing to the more extensive WMH on conventional MRI. Nevertheless, we found no significant differences exist in fiber length and volume of the four subcomponents of SLF between groups. This may be due to the fact that although the white matter structural integrity is injured in CSVD patients in the early stage of the disease, the white matter fiber tracts are not entirely destroyed, and to a certain extent, the original morphological measures, such as length and volume, are maintained. The MD, AD, and RD values of bilateral AF and left SLF-III were positively correlated with the WMH volume of patients. This also indicates that the more severe the WMH, the more obvious the structural integrity injury of bilateral AF and left SLFIII, which is consistent with the findings based on visual assessment of WMH load.

Previous studies have demonstrated that the disruption of SLF structural integrity is associated with cognitive decline in CSVD study participants [24, 25, 43–46]. However, SLF is a complex fiber tract and previous studies failed to investigate the injury of which subcomponent is closely related to cognitive decline in CSVD study participants. In the current study, MMSE and MoCA were applied for global cognitive assessment. They are known as classic cognitive testing scales, which are easy to operate and widely used in clinics [47, 48]. When we further analyzed the SLF subcomponents, we found that the increased MD value of left SLFIII was correlated with the study participants’ global cognitive decline. A growing number of studies have found that each SLF subcomponent owns unique function [23, 49]. The left SLFIII is mainly involved in language function such as articulatory processing and auditory language comprehension, which belong to the category of cognition [50]. Language function is an important part of cognitive domains [51]. Language dysfunction in CSVD patients has also been demonstrated in some studies [52–54]. Although early language dysfunction is easily ignored in clinical practice, it has a certain impact on patients’ global cognitive function [51]. When the injury of the structural integrity of the fiber tract intensifies, the patient’s cognitive function will also decline. This may be the consequence of structural injury affecting specific cognitive domains, or it may result from the disruption of cognitive pathway connectivity that impacts global cognition. Previous studies have demonstrated that the role of site-specific WM structural integrity injury in cognitive effects in CSVD study participants applies not only to isolated cognitive domains but also to global cognitive function [55]. By this token, the structural integrity injury of left SLFIII correlates to the global cognitive function of CSVD study participants.

There exist some limitations in this study. First, as a cross-sectional study, we could not understand the changes in white matter structural characteristics and cognitive function of each CSVD patient over time. Second, no health control group was set up in this study. CSVD is known as an age-related disease. Study participants at least 55 years old were enrolled in the current study. In addition, WMH can be detected in more than 70% of the population over the age of 60 [56]. Consequently, setting up a wholly healthy and age-matched control group is challenging. In future studies, the age range of subjects will be expanded gradually. Third, although MoCA is a classic and widely used cognitive assessment test, we only used it to assess subjects’ global cognitive function. For the impairment of each cognitive domain, we need to conduct a more detailed assessment through advanced neuropsychological testing, such as auditory language learning test, trail making test, and verbal fluency test. In future studies, we will assess all subjects using a complete set of cognitive assessment scales. At last, we only investigated the alterations in structural integrity of SLF subcomponents with increasing WMH severity as well as the relationship to cognitive performance in CSVD in the current study. Previous studies have shown that WMH are likely related to secondary degeneration and are related to cognitive performance [57]. As for whether alterations in structural integrity of SLF subcomponents are due to secondary degeneration, such as the presence of lacunes in the concerned bundles, and are related to cognitive function, we will explore this in future studies. In addition, previous studies have found that brain atrophy and lacunes are associated with cognitive dysfunction in CSVD patients [58, 59]. Tian’s results indicated that the DTI index had substantial relationships with WMH, lacunes, and brain atrophy [60]. These are very valuable lines of research. We will also explore the relationship between brain atrophy or lacunes and cognitive dysfunction and diffusivities in depth in the future.

In the end, new version eddy greatly improves the quality of data correction [61, 62], especially distortion correction, slice-to-volume movement correction and susceptibility-by-movement interactions correction etc. Since no additional set of opposite phase encoding direction B0 images were obtained in our experimental sample to complete topup correction, the tool of eddy could not be used to satisfy the analysis which can further improve the analysis accuracy. But we have taken steps to improve the analysis accuracy of eddy correct. In the EPI sequence, the larger the TE value and the longer interval time of echo spacing, the more significant the deformation of phase encoding direction is. Therefore, we adjusted and optimized the sequence protocol to minimize the impact of machine parameters on image distortion. In addition, we performed manual and automatic quality control protocol with DTIprep(v1.2.11) before DTI preprocessing and excluded the data with obvious slice-to-volume movement by manually check. Generally, susceptibility differences at the air-tissue boundary, such as the prefrontal lobe, anterior temporal lobe and anterior skull base, lead to alterations of the B0 field that can cause spatial displacements of several pixels. Our current findings are located in the deep brain, not in the above areas, and may receive less interference from deformation. In future studies, we will refine the image acquisition and use the state-of-the-art eddy tool for correction.

## Conclusion

The structural integrity injury of bilateral AF and left SLFIII is more severe with the aggravation of WMH. The structural integrity injury of left SLFIII correlates to cognitive impairment in CSVD.

### Electronic supplementary material

Below is the link to the electronic supplementary material.

Supplementary Material 1

## Data Availability

The datasets in this study are available from the corresponding author on reasonable request.

## Abbreviations

AD: Axial diffusion
AF: Arcuate fasciculus
CSVD: Cerebral small vessel disease
DTI: Diffusion tensor imaging
FA: Fractional anisotropy
FDR: False discovery rate
FLAIR: Fluid attenuation inversion recovery
MD: Mean diffusion
MMSE: Mini-mental State Examination
MoCA: Montreal Cognitive Assessment
RD: Radial diffusion
SLF: Superior longitudinal fasciculus
VCI: Vascular cognitive impairment
WM: White matter
WMH: White matter hyperintensity
