# Supplementary material for: Alterations in structural integrity of superior longitudinal fasciculus III associated with cognitive performance in cerebral small vessel disease
Source: BMC Med Imaging. 2024 Jun 10;24:138. doi: 10.1186/s12880-024-01324-2 (PMC11165890; doi:10.1186/s12880-024-01324-2)
Supplement: Supplementary file 1 — Supplementary Material 1 [file 12880_2024_1324_MOESM1_ESM.docx]

Supplementary material

1. **Data analysis code excerpts**

#! /bin/bash

#part01, convert & eddy & dtifit

dti_path=/csvd_demo/dti

t1_path=/csvd_demo/t1

dcm2niix -f dwi -z y -o /csvd_demo ${dti_path}

dcm2niix -f t1 -z y -o /csvd_demo ${t1_path}

eddy_correct dwi.nii.gz data.nii.gz 0

fdt_rotate_bvecs data.bvec bvecs *.ecclog

cp -v data.bval bvals

dwiextract data.nii.gz - -bzero | mrmath - mean b0_mean.nii.gz -axis 3

bet b0_mean.nii.gz nodif_brain -f 0.29 -m

dtifit -k data.nii.gz -o dti -m nodif_brain_mask.nii.gz -r bvecs -b bvals --save_tensor

cp -v dti_L1.nii.gz dti_AD.nii.gz

fslmaths dti_L2.nii.gz -add dti_L3.nii.gz -div 2 dti_RD.nii.gz

#part02, image registration

fslreorient2std t1.nii.gz re_t1.nii.gz

bet re_t1.nii.gz re_t1_brain -f 0.29 -m -S -B -v

fslreorient2std nodif_brain.nii.gz re_nodif_brain.nii.gz

mkdir -p xfms

flirt -in re_nodif_brain.nii.gz -ref re_t1_brain.nii.gz -omat xfms/diff2str_linear.mat -dof 9 -out

xfms/diff2str_linear_warped.nii.gz

convert_xfm -omat xfms/str2diff_linear.mat -inverse xfms/diff2str_linear.mat

flirt -in re_t1_brain.nii.gz -ref $FSLDIR/data/standard/MNI152_T1_1mm_brain.nii.gz -omat

xfms/str2standard_linear.mat -dof 12 -out xfms/str2standard_linear_warped.nii.gz

convert_xfm -omat xfms/standard2str_linear.mat -inverse xfms/str2standard_linear.mat

convert_xfm -omat xfms/diff2standard_linear.mat -concat xfms/str2standard_linear.mat

xfms/diff2str_linear.mat

convert_xfm -omat xfms/standard2diff_linear.mat -inverse xfms/diff2standard_linear.mat

fnirt --in=re_t1_brain.nii.gz --aff=xfms/str2standard_linear.mat --

ref=$FSLDIR/data/standard/MNI152_T1_1mm_brain.nii.gz --

refmask=$FSLDIR/data/standard/MNI152_T1_1mm_brain_mask_dil --

config=T1_2_MNI152_2mm --cout=xfms/str2standard_nolinear_warp --

iout=xfms/str2standard_nolinear_warped.nii.gz

invwarp -w xfms/str2standard_nolinear_warp -o xfms/standard2str_nolinear_warp -r

re_t1_bias_brain.nii.gz

convertwarp -o xfms/diff2standard_nolinear_warp -r

$FSLDIR/data/standard/MNI152_T1_1mm_brain.nii.gz -m xfms/diff2str_linear.mat -w

xfms/str2standard_nolinear_warp

convertwarp -o xfms/standard2diff_nolinear_warp -r re_nodif_brain.nii.gz -w

xfms/standard2str_nolinear_warp --postmat=xfms/str2diff_linear.mat

applywarp --in=re_nodif_brain.nii.gz --out=xfms/diff2standard_nolinear_warped.nii.gz --

ref=$FSLDIR/data/standard/MNI152_T1_1mm_brain.nii.gz --

warp=xfms/str2standard_nolinear_warp --premat=xfms/diff2str_linear.mat

#part03, bedpost & xtract by GPU version

bedpostx_datacheck /csvd_demo

bedpostx_gpu /csvd_demo

cp -vr xfms/* /csvd_demo.bedpostX/xfms

xtract -bpx /csvd_demo.bedpostX -out /csvd_demo.bedpostX/xtract -species HUMAN -stdwarp /csvd_demo.bedpostX/xfms/standard2diff_nolinear_warp.nii.gz /csvd_demo.bedpostX/xfms/diff2standard_nolinear_warp.nii.gz -gpu

xtract_viewer -dir /csvd_demo.bedpostX/xtract -species HUMAN

xtract_stats -d /csvd_demo/dti_ -xtract /csvd_demo.bedpostX/xtract -w

/csvd_demo.bedpostX/xfms/diff2standard_nolinear_warp.nii.gz -r /csvd_demo/dti_FA.nii.gz -

means vol,prob,length,FA,MD,AD,RD

echo "Jobs done"

1. **MRI processing figures**


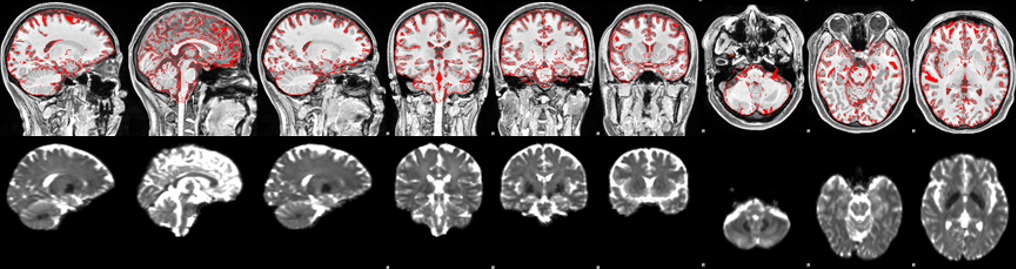


Figure S1 The results and images of DTI linear registration to 3D-TIWI.


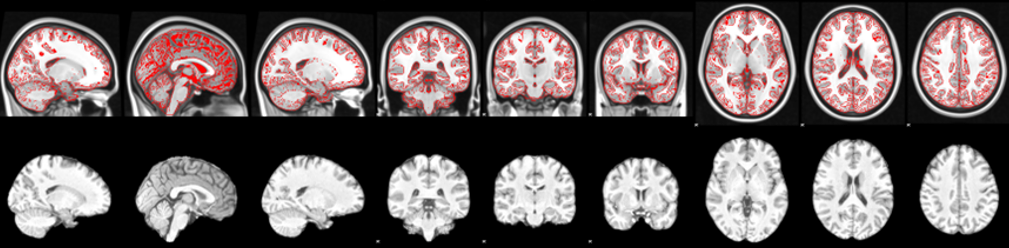


Figure S2 The results and images of 3D-TIWI nonlinear registration to standard space MNI152.


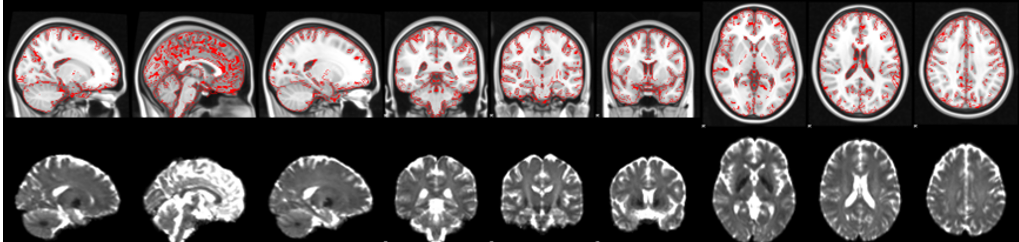


Figure S3 The results and images of DTI nonlinear transformation to standard space MNI152 by 3D-TIWI.


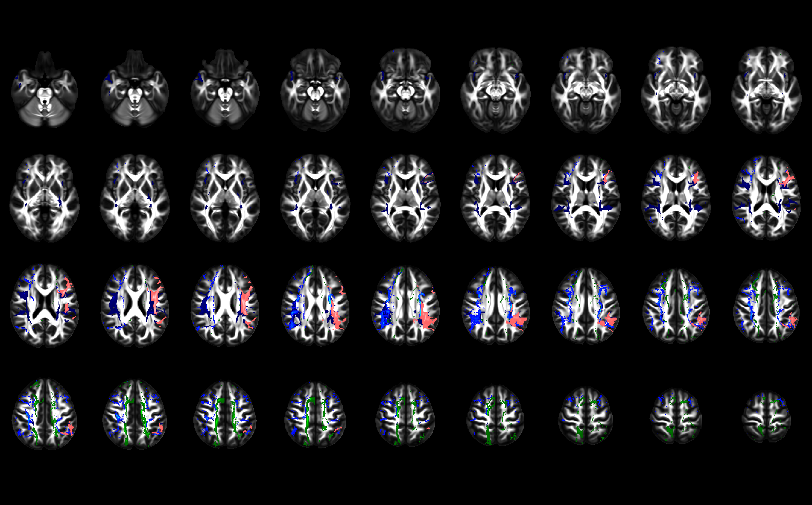

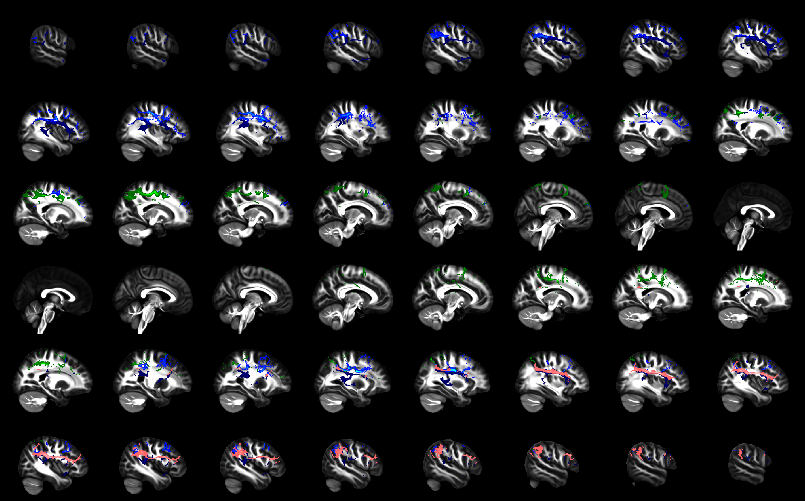


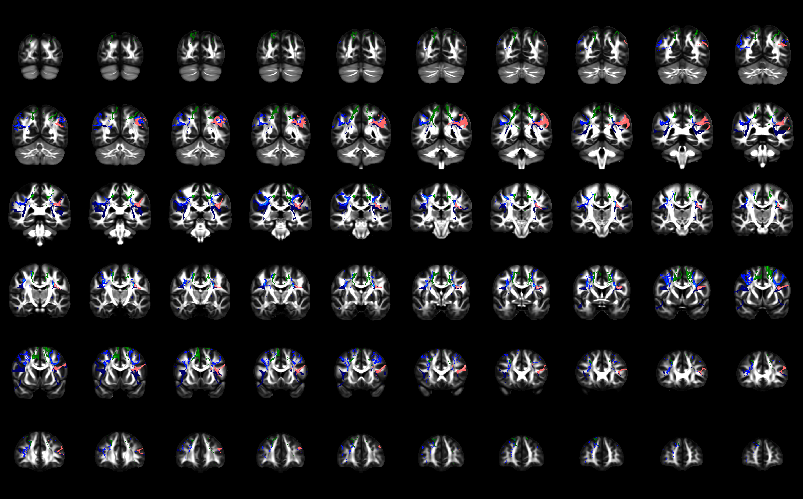


Figure S4 The bilateral AF, SLFI, SLFII, and SLFIII.

1. **ARWMC score**

The age-related white matter changes (ARWMC) scale was used to evaluate the degree of white matter lesions in five brain regions, namely the frontal, parieto-occipital, temporal, infratentorial/cerebellum, and basal ganglia regions. The left and right hemispheres were scored respectively, with a total score of 30 points. The definitions of rating scores (0–3) are shown in the Table S1 below. In this study, 110 patients had a median ARWMC score of 6, with interquartile range of 3 to12. Table S2 shows the distribution of white matter hyperintensity in study participants.

Table S1 The ARWMC Rating Scale

| White matter lesions | |  |
| --- | --- | --- |
| 0 | No lesions (including symmetrical, well-defined caps or bands) | |
| 1 | Focal lesions | |
| 2 | Beginning confluence of lesions | |
| 3 | Diffuse involvement of the entire region, with or without involvement of U fibers | |
| Basal ganglia lesions |  | |
| 0 | No lesions | |
| 1 | 1 focal lesion (≥5 mm) | |
| 2 | ＞1 focal lesion | |
| 3 | Confluent lesions | |

Table S2 Distribution of white matter hyperintensity in study participants

| Distribution | N (%) |
| --- | --- |
| Frontal | 100 (90.9) |
| Parieto-Occipital | 79 (71.8) |
| Temporal | 50 (45.5) |
| Infratentorial/Cerebellum | 19 (17.3) |
| Basal Ganglia Regions | 48 (43.6) |

1. **Automatic extraction and volume calculation of WMH**

Wisconsin White Matter Hyperintensities Segmentation Toolbox (W2MHS) (http://www.nitrc.org/projects/w2mhs) was used to automatically mark and extract the volume of the white matter hyperintensity. It is a WMH segmentation toolkit developed by the Wisconsin Alzheimer Research Center. It consists three modules. (1) Preprocessing module: Firstly, 3D TIWI and 3D FLAIR data were registered to standard space, and partial volume estimates of white matter, gray matter and cerebrospinal fluid were extracted based on SPM12. (2) Segmentation module: Using a classifier based on random forest and support vector mechanisms to detect the distribution of white matter lesions; (3) Quantitative module: Calculate the number of voxels occupied by white matter lesions, then multiply by the voxel resolution of FLAIR images to obtain the WMH volume (mm^3^), and finally obtain the WMH volume of periventricular, deep white matter and whole brain.

We used partial correlation analysis to explore the relationship between WMH volume and DTI-derived indexes, with age, sex, and education as covariates. The results showed that MD, AD, and RD values of bilateral AF and left SLF-III were positively correlated with WMH volume of patients (p < 0.05, FDR corrected). See Table S3 for details. This indicates that the structural integrity injury of bilateral AF and left SLFIII was significantly more severe with the aggravation of WMH, which is consistent with the findings based on visual assessment of WMH load.

Table S3 Correlation between DTI-derived indexes and the WMH volume

| Tract | MD | | AD | | RD | |
| --- | --- | --- | --- | --- | --- | --- |
|  | r | p | r | p | r | p |
| AF-L | 0.537 | 0.001 | 0.580 | ＜0.001 | 0.479 | 0.002 |
| AF-R | 0.566 | ＜0.001 | 0.594 | ＜0.001 | 0.510 | 0.001 |
| SLFIII-L | 0.363 | 0.025 | 0.396 | 0.014 | 0.338 | 0.038 |
